# Nordic Orofacial Test-Screening Protocol as a Tool for Assessment of Orofacial Dysfunction in Pediatric and Adult Patients

**DOI:** 10.3390/diagnostics15131656

**Published:** 2025-06-29

**Authors:** Karolina Szuflak, Karolina Gerreth, Maurycy Jankowski, Roksana Malak, Włodzimierz Samborski, Michał Karlik

**Affiliations:** 1 Department of Phoniatrics and Audiology, Poznan University of Medical Sciences, 60-355 Poznan, Poland; 2 Doctoral School, Poznan University of Medical Sciences, 60-812 Poznan, Poland; 3 Department of Risk Group Dentistry, Chair of Pediatric Dentistry, Poznan University of Medical Sciences, 60-812 Poznan, Poland; karolinagerreth@ump.edu.pl; 4 Greater Poland Center of Digital Medicine, Poznan University of Medical Sciences, 60-806 Poznan, Poland; mjankowski@ump.edu.pl; 5 Department of Histology and Embryology, Poznan University of Medical Sciences, 61-781 Poznan, Poland; 6 Department and Clinic of Rheumatology, Rehabilitation and Internal Medicine, Poznan University of Medical Sciences, 61-545 Poznan, Poland

**Keywords:** oral health, masticatory function, malocclusion, multidisciplinary cooperation, musculoskeletal diseases, diagnostics, screening

## Abstract

**Background/Objectives**: The Nordic Orofacial Test-Screening (NOT-S) is a versatile tool used for the assessment of orofacial function. The aim of this study was to introduce the Polish version of the NOT-S along with the results of its cultural adaptation. **Methods**: The original NOT-S was validated into the Polish language in accordance with the current questionnaire translation standards, including the cultural adaptation, which consisted of an examination of Polish society. The pilot study, as a cultural adaptation, was carried out among fifty people between the ages of 3 and 34 (Mean—15.8, SD—8.9). **Results**: During the translation, three word discrepancies were noted, and also many linguistic equivalences, such as semantic, empirical, conceptual, and other differences, were reported. The average total NOT-S score during cultural adaptation was 1.62 (SD 1.16; Range 0–5). Abnormalities in section III, i.e., habits (78%), were found most frequently in the subjects. A thorough data analysis showed statistically significant results in section IV of the interview, i.e., chewing and swallowing (*p* = 0.00073), and also significant results in section 3 of the examination, i.e., facial expression (*p* = 0.00006). **Conclusions**: The Polish version of the NOT-S is linguistically comprehensible and culturally equivalent, and can be used for the examination of children, adolescents, and adults. The findings of this study indicate that orofacial function screening is advisable in the healthy population.

## 1. Introduction

Orofacial functions are the result of the complex and integrated actions of the central nervous and neuromuscular systems [1]. They comprise a range of crucial activities, such as breathing, chewing, and swallowing, as well as engaging in social interactions through speech and emotion expression [2]. Impaired orofacial function, or dysfunction, can therefore impair daily activities in many areas of life. Etiological factors of orofacial dysfunction can include both congenital and acquired diseases [3,4,5,6,7,8,9,10].

The Nordic Orofacial Test-Screening (NOT-S) is a screening tool developed to assess or screen for orofacial dysfunction in children aged 3 years and older, adolescents, and adults. NOT-S was developed in 2007 by Bakke et al. as part of the Scandinavian project of the Nordic Society for Disability and Oral Health (NFH) [2]. It is recommended for the diagnosis of patients with speech, chewing, and swallowing difficulties, as well as a range of other orofacial function impairments. A crucial advantage of the tool is its suitability to be used by a variety of professionals, including doctors, dentists, physiotherapists, and speech therapists. Initially, the test was created in Swedish, and it was later translated into other Nordic languages and English [2]. Currently, the NOT-S, accompanied with an illustrated guide, is available online [11] in the following versions: French, German, Italian, Spanish, Portuguese, Brazilian Portuguese, Japanese, Chinese, Croatian, Czech, Estonian, Latvian, Romanian, Slovakian, Serbian, and Turkish. However, to date (as of 27 May 2025), it has not been officially translated into Polish and uploaded to a publicly accessible website.

The NOT-S can be used as a standard tool for assessment of orofacial function, evaluating the results of oral rehabilitation, and facilitating comparability of results obtained by researchers during ongoing studies [12]. Due to the complexity of most of the orofacial abnormalities, including the presence of manifestations of chronic systemic diseases within the stomatognathic system, the approach to the diagnosis of patients with masticatory dysfunction should always be interdisciplinary. According to literature reports, the results of studies conducted using the NOT-S assessment are closely correlated with the presence of temporomandibular joint dysfunction [12] and nocturnal bruxism [13]. In addition, the Nordic Orofacial Test-Screening has also been used during studies of orofacial function abnormalities in patients with nervous system disorders, including cerebral palsy, Parkinson’s disease, amyotrophic lateral sclerosis, and multiple sclerosis [7,8,9,10,14,15,16]. Many genetically determined syndromes are associated with the onset of difficulties in eating, speaking, or breathing. Hence, the NOT-S can also be a very helpful tool in examining patients with, e.g., Treacher–Collins syndrome, Prader–Willi syndrome, ectodermal dysplasia, or Down syndrome [3,4,5,6,17].

It should be mentioned that there are two other tests through which stomatognathic changes can be assessed, the Protocol of Orofacial Myofunctional Evaluation With Scores (OMES) and Research Diagnostic Criteria for Temporomandibular Disorders (RDC/TMD). The former serves as a tool for assessing orofacial function, and has not been translated into Polish to date. Its advantage is that it can be used to examine children. However, it lacks items relating to sensory function and salivation [18]. The latter test, the RDC/TMD, allows the evaluation of functional disorders of the masticatory musculoskeletal system, with a highly detailed assessment of temporomandibular joint dysfunction. It has been subjected to translation and validation into Polish, but items on sensory function, breathing, or salivation were not included. An additional limitation is that the examination using the RDC/TMD protocol can only be performed by a dentist [19].

The lack of a publicly available tool in Polish, such as the NOT-S, prompted the researchers to consider the need to create a new instrument, or to translate, adapt, and validate an existing questionnaire. As with other such documents, developing a new test that includes a patient interview and a clinical examination section would have involved high costs, longer time, and the limitations of not being able to compare results with data obtained for patients studied in other countries [20]. Hence, the second alternative seemed more economical, practical, and efficient. However, it is necessary to conduct a cultural adaptation of the test. That process involves translating a given tool into Polish and adapting it to the conditions of Polish culture. Then, the equivalence of both versions was examined, their accuracy was assessed, and the psychometric parameters of the final version of the tool were determined [21].

In Poland, the problem of stomatognathic abnormality appearance is relatively common, with a high percentage of patients and abnormality incidence compared to the world population [22]. Hence, the need for the NOT-S to implement accurate diagnosis of orofacial function among the Polish population was identified. Our hypothesis was that in the Polish population, orofacial dysfunctions exist. However, to enable the use of the tool in Poland, it is necessary to translate, adapt, and validate it. This process should be conducted considering commonly existing international rules to ensure that the new language version can be used to conduct research, the results of which can be compared with those obtained by researchers from other countries [23].

The aim of the study was to introduce the Polish version of the Nordic Orofacial Test-Screening, along with a presentation of the successive stages of translation from English to Polish, and an analysis of the results relating to the cultural adaptation of the Polish language version.

## 2. Materials and Methods

The Nordic Orofacial Test-Screening consists of two parts, a structured questionnaire interview and a clinical examination, with six sections each. The interview includes questions relating to sensory function, breathing, habits, chewing and swallowing, excessive salivation, and dryness of the mouth (I–VI). On the other hand, the clinical examination assesses the face at rest, nasal breathing, facial expression, mandibular and masticatory muscle function, oral motor function, and speech (1–6). Some sections have subsections, for example, (3A), (3B), and (3C). NOT-S is accompanied by an illustrated guide used during the study, which includes a set of photos or pictures that allow a more detailed description of the activities the subject is asked to perform [2]. When conducting the questionnaire interview, the patient or their parent/guardian is obliged to answer in an unambiguous manner (YES/NO). If a disorder is present, the researcher writes an “X” in the box next to the question. On the other hand, if the abnormality is not present, a “0” is input in the box. If the patient cannot do a movement, for example, close the mouth while taking five breaths (2A), in the test, there are special indications for examination, like in this situation, help and close the patient’s lips by hand. If it is impossible to assess a given activity for various reasons, or the examined subject is not able to answer the question, one should mark “-” in the box. In a situation where, in a given section, there is one or more “X” answers, then “1” should be input in the rightmost box. The final score is the sum of scores from all sections and ranges from 0 to 12, with higher values indicating the presence of more orofacial dysfunctions [2].

### 2.1. Methods

The translation of the Nordic Orofacial Test-Screening into Polish received official written permission from its authors. The entire procedure related to the translation of the document was performed in accordance with generally accepted principles described in the professional literature and included a translation into Polish, back translation into English, and cross-cultural adaptation [21,24,25,26].

The procedure of translation of the NOT-S from the official English-language version into Polish was undertaken by a team of three people, including two bilingual medical professionals (a dentist and a physiotherapist), as well as a professional translator. The resulting three versions of the document in Polish were then compared, and a single study was determined by consensus, which was back-translated by another professional translator who had graduated from a medical school in the United Kingdom. This person had not previously read the original version of the NOT-S in English. This stage is known as blind back-translation. At the next stage, the obtained English-language version was compared word by word with the official NOT-S in English. This procedure was carried out, in a joint meeting, by all the specialists and translators who participated throughout the validation process. As a result, according to the guidelines, errors in translation, interpretation, or typography in the target Polish language version of the document were corrected. In addition, it was decided to subject the final variant of the NOT-S to consultation with professionals, i.e., a speech therapist and a medical biologist specializing in normal anatomy, whose native language is Polish, to determine the correctness of the nomenclature contained in the NOT-S text. Cultural adaptation, as a pilot study, was carried out by using a Polish version of NOT-S among Polish volunteers, with notes taken if some questions or comments were unclear. It was conducted by a professional with a master’s degree in physiotherapy. The authors have permission to use this instrument from the copyright holders.

### 2.2. Materials

The study was approved by the Poznan University of Medical Sciences Bioethical Committee (resolutions 804/20, 482/21, and 874/22).

The cultural adaptation was conducted from June to December 2022 in a group of fifty subjects, women and men (women—30, mean age—16.4, men—20, mean age—15), aged 3 to 34 (Mean—15.8, Median—15.5). Participants in the study were recruited through advertisements at health care entities in the Greater Poland Area: the Health Center in Sroda Wielkopolska and the Health Center in Poznan; among patients of the University Center for Dentistry and Specialized Medicine in Poznan, as well as among students of the Poznan University of Medical Sciences majoring in medicine and dentistry; and at the Elementary School No. 2 in Kornik. The directors of all the above-mentioned establishments agreed to conduct the study. This study included participants without structural changes in the craniofacial region. The exclusion criteria comprised the presence of disorders of the subjects’ nervous system, genetic syndromes, or genetically determined diseases, and patients after facial trauma or with chronic diseases that could affect orofacial function. Written informed consent was requested from all participants, who were provided with information about the study prior to participation. In the case of subjects under the age of 18, the data were obtained from their parents or legal guardians, who consented to perform the procedure on their children. During the examination of minors, parents or guardians were present to help answer the questions correctly in case of doubt, for example, with the answer regarding snoring at night. All subjects or accompanying persons were asked for their opinions on the level of understanding of the manual, their assessment of the unambiguity of the instructions given, or any comments on their content.

### 2.3. Statistical Analysis

The data obtained were subjected to statistical analysis using the PQStat software (GEN 4.5.10). The chi-square test was used to determine statistically significant correlations between the results of the pilot study scores within sections (1–6 and I–VI) of NOT-S. The statistical comparison was within each section separately, e.g., within section (3) using results from subsections (3A), (3B), and (3C). Results with *p* ≤ 0.05 were considered statistically significant.

## 3. Results

### 3.1. Validation Part

Table 1 presents three errors, referred to as discrepancies, that were found between the original NOT-S in English and the back translation presented. In addition, there were linguistic equivalences, such as semantic, experiential, conceptual, and other discrepancies, related to standard or adopted language use. It is shown in Table 2. No idiomatic or vocabulary equivalences were observed.

### 3.2. Cultural Adaptation

The results on cultural adaptation showed that the Polish version of the NOT-S was correctly understood by those taking part and/or the parents of the examined children. No phrases were recorded that were not understood or misunderstood. There were, however, difficulties in fifteen people (30% of the total) in answering questions that included the statement “everyday”.

During the examination, the mean total NOT-S score of 1.62 (SD 1.16; Range 0–5) was recorded in Table 3. The lowest score observed was 0.0, with the highest value of 5.0.

Table 4 shows that a final NOT-S score of 0.0 was given to eight subjects (16% of the total), 1.0 appeared among sixteen subjects (32%).of the total), followed by 2.0 in eighteen participants (36% of the total), 3.0 in four subjects (8% of the total), 4.0 in three (6% of the total), and one person was given a score of 5.0 (2% of the total).

Table 5 shows the results by section. The most common abnormality in the questionnaire section was related to the “habits” section, and amounted to thirty-nine points, accounting for 78% of the total number of participants presenting at least one of the listed abnormalities. In addition, the main emerging disorder in this section among the subjects (44% of the total) was sucking or biting the lip, tongue, or cheeks. On the other hand, in the examination section, the most common emerging abnormality was asymmetric masseter muscle action, observed in 14% of the total subjects.

Statistical analysis showed significant differences between the results relating to the part of the questionnaire within section IV “chewing and swallowing” (*p* = 0.00073), and within part 3 of the test “facial expression” (*p* = 0.00006) (Table 5).

## 4. Discussion

The study presents the Polish language version of the Nordic Orofacial Test-Screening (NOT-S), the description of the process of the translation and validation of this assessment, and the results of the cultural adaptation, as a pilot study, using internationally accepted methodology procedures [in the Supplementary Materials]. That assessment will be valuable to many healthcare providers to find those individuals who need further clinical examinations, treatment interventions, or adaptations in the orofacial area. All patients participating in the pretesting phase reported understanding the items. This suggests that the adaptation was auspicious [22].

The translation and validation process showed many versions to convey the same meaning of the words, but also indicated how important details are, while testing within patients to make the most accurate results. In this study, that step of translation and validation was carried out, with the agreement and understanding of all specialists, while the suitable word was chosen. During the cultural adaptation, the most common doubt that arose was the statement, “every day”, in the questionnaire section, as there were times when respondents performed an activity frequently but were not sure if it took place every day. Similar observations to those presented in the paper were obtained by Leme et al. during a cultural adaptation of NOT-S into Brazilian Portuguese [27]. The authors conducted the NOT-S study in a group of twenty people, aged 8 to 14, and noted doubts about the phrase “almost every time”, which was initially translated literally. However, after the survey was performed and the responses analyzed, it was decided to change the phrase to “muitas vezes” (English for “often”) [27].

In the clinical part of the present study, it was noted that as many as 44% of the subjects had difficulty maintaining seriousness when observing an illustration showing a hen with chicks in the “sensory and function” section. Sometimes, the patient did not understand that he or she should only look at the illustration. On more than one occasion, comments or statements such as “Chicken”, “That’s a chicken”, or asking what action should be performed (“...but what should I do?”) would be made, or patients would start laughing or smiling, making it difficult to assess the face at rest. The researcher in such a situation asked the patient to stare at a single point far away in space and make an assessment referring to the “face at rest” section (1). The problems observed during the subjects’ performance of the task using an illustration depicting a hen make us consider whether citizens of other nationalities are also cheered up by the image of this animal. It can be assumed that replacing this illustration with a generic scene with many details would mobilize the patient to observe carefully and better facilitate the aim of the study. While conducting the present study, it was also observed that there may have been an unclear message in the command (3A) “close your eyes tightly”. It happened that some subjects only gently closed their eyelids, and did not tighten them as illustrated in the photo of the woman’s face included in the guide. It was only after the examiner’s suggestion that the subject tighten their eyes that the desired effect was achieved.

The first report of a study performed using the NOT-S assessment was published in 2007 by its authors, namely, Merete Bakke, Birgitta Bergendal, Anita McAllister, Lotta Sjögreen, and Pamela Åsten [2]. One hundred and twenty individuals with various types of genetic, congenital, or acquired orofacial defects, as well as chronic disorders and diseases or developmental delays, were analyzed. The study population included sixty-one women (50.8%) and fifty-nine men (49.2%), aged 3 to 86 (Mean—26.3, Median—18.0). Sixty generally healthy participants were included in the control group, with thirty-five (58.3%) women and twenty-five (41.7%) men, aged 3 to 78 (Mean—30.4, Median—31.5) [2]. In the study group, the mean total NOT-S score was 4.1 (SD 2.6; Median 4.0; Range 0–10), while in the control group it was 0.4 (SD 0.7; Median 0.0; Range 0–2). In addition, in the healthy group, a score of 0.0 was shown in thirty-eight subjects (63% of the total), 1.0 among eighteen subjects (30% of the total), and 2.0 in four subjects (7% of the total). A comparison of the results obtained by Bakke et al. [2] for the control group with those obtained during cultural adaptation in the present study for a population of healthy individuals, indicates that the highest score of the total NOT-S score in the Scandinavian researchers was 2.0, while it was as high as 5.0 in the study performed in the Polish population. In addition, in the present study, 16% of subjects were observed to have a score above 2.0. Bakke et al. [2], when conducting the assessment, most frequently noted disorders related to breathing and habits (11.7% of the total), while in the clinical examination section, changes were observed primarily in the “face at rest” section and affected 6.7% of the total study group. Similar observations were made in this study, as most of the abnormalities found on the basis of the NOT-S were noted in the “habits” section (78% of the total). On the other hand, in the “masticatory muscle and jaw function” section of the survey, asymmetrical muscle tension distribution was mainly diagnosed in the Polish population, affecting 14% of the examined subjects. These findings are worth examining further, as they may suggest, for example, a greater exposure of Poles to stress. The period in which the study was conducted could be important in that regard. It is likely that the timing of the Corona Virus Disease 2019 pandemic (COVID-19) may have had a significant impact, resulting in children and adolescents more frequently exhibiting adverse habits, which, according to numerous reports, are often associated with increased stress [28,29,30,31,32], even though the higher tension distribution in the masticatory muscles can be caused by parafunctional habits like sleep bruxism or an uneven distribution of occlusal contacts [13,33,34].

The authors who developed a Brazilian version of the Portuguese variation of the NOT-S tool surveyed a group of three hundred and twenty-eight students in four randomly selected public schools in the city of Piracicaba [35]. Participants were between the ages of 8 and 14, of which one hundred and ninety-seven were girls (60.1% of the total), while one hundred and thirty-one were boys (39.9% of the total). The NOT-S was used by the researchers to divide children and adolescents into subgroups of students without habits and with habits for further analysis of the results obtained. It was found that habits occurred in 71.3% of the children, and this result proved to be statistically significant between the above subgroups (*p* = 0.0001). In addition, habits were more common among girls (62.8% of children), compared to boys (*p* = 0.001). In the subgroup of those with inappropriate habits, the average total NOT-S score was 3.0 ± 1.4, while among the habit-free group, the value was lower at 2.0 ± 1.3. It is worth noting that the authors found a statistically significant difference in both subgroups between the sexes (*p* < 0.001) [35]. Comparing these results to those obtained in the present research, one may note a higher percentage of habit-exhibiting participants (78%) than in the study by Leme et al. [35] (71.3%). Therefore, it can be concluded that such disorders are more common in the population of children, adolescents, and young adults in Poland.

A project based on a younger group of participants was conducted in 2019 at the Pediatric Dentistry Department of the Faculty of Dentistry, Hacettepe University in Turkey, with the results published by Ozturk et al. [36]. The aim of the study was to evaluate the orofacial function and oral health of healthy children and determine the potential correlations between these aspects. Four hundred children between the ages of 5 and 8 (Mean 6.5 years old) were examined dentally and with the help of, e.g., the NOT-S assessment, with the mean total NOT-S score of 3.45 ± 1.79. In comparison, the score received in a group of Polish individuals was significantly lower and amounted to 1.62 ± 1.16. The most frequently observed abnormality during the NOT-S examination conducted by Ozturk et al. [36] turned out to be habits, which were presented by 51.0% of the total subjects, with this value turned still lower than the one detected in the presented study (78% of the total). On the other hand, the results obtained in the “chewing and swallowing” section were 45.3% for the total participants of the Turkish study, notably higher than in received results (26%) in section IV. In addition, in the clinical examination section, the Turkish team recorded the highest percentage of pathology in the “facial expression” section (49.3% of the total subjects), while in this study, the results in section 3 had a much lower value, as only 6% of the participants were affected. It is also important to note the authors’ statement that there have been no previous studies on orofacial myofunctional disorders and oral health among healthy populations [36]. Since then, a study in a similar group of participants was carried out by Abd-Elsabour et al. [37]. The results of the orofacial diagnosis conducted both by numerous researchers and our team prove that abnormalities in this area affect healthy people in a high percentage. Hence, it can be concluded that screening among this population is warranted and needed for prompt diagnosis and further treatment.

An analysis of the available literature relating to the assessment of orofacial function in healthy individuals using Nordic Orofacial Test-Screening, as well as the results of the present research, indicate that the presence of unfavorable habits affects a high percentage of children and adolescents living in countries located on different continents, including Europe [2,38], South America [35] and Asia [36]. Thus, there is a need for measures to help counteract the emergence of habits or manage them in this patient population.

Although the presented test (NOT-S) is a relatively simple and inexpensive tool for diagnosis of orofacial function in patients, so far, no official validation has been carried out for the Polish language version and posted on a publicly available website [11], from which it is possible to download the NOT-S free of charge in various language versions. Hence, one may assume that the procedure carried out will contribute to the widespread use of the tool both by professional practitioners in various fields of medicine, as well as researchers, to conduct studies relating to this issue. Moreover, a great advantage of this instrument is also the fact that NOT-S can be used by representatives of various medical specialties, including speech therapists [2,3,15], dentists [4,13,36,39,40], neonatologists [41], and occupational therapists [42]. There are also reports in which the field of the professional who conducted the study was not clearly specified [10,14,29,34,37,38,43,44,45,46,47,48,49,50,51]. The limitation of our examination was the failure to include elderly people, for the reason of apprehension about many symptoms in these patients, which can influence the orofacial functions. This subject can be an area for future research, as well as an increase in the number of subjects.

In conclusion, it is worth noting that the Nordic Orofacial Test-Screening (NOT-S) has so far also been used to evaluate orofacial changes among patients with various disease entities, such as genetic defect syndromes [2,3,4,5,6,46,52], neurological [8,9] or neurodevelopmental disorders [14,43], respiratory diseases [44], speech defects [45], and stomatognathic lesions [53] or leukemia [47]. According to the researchers’ assessment, the NOT-S is easy to use and can be applied to identify orofacial dysfunctions and refer patients for further diagnosis or treatment. Moreover, the NOT-S assessment serves as a screening tool and should be used to assess dysfunctions that require further examination or referral for more specialized testing [12]. In addition, the answers given by the respondents to the items in the NOT-S were used to analyze the results, dividing the participants into subgroups [35,37]. It is worth mentioning that the NOT-S can also be useful for evaluating abnormalities in the oral cavity, or for assessing the effects of rehabilitation within this region. The use of the Nordic Orofacial Test-Screening provides an opportunity to compare the results obtained in scientific studies and apply them during daily clinical practice [12]. In addition, it should be mentioned that the diagnosis using the NOT-S assessment can be obtained in a relatively short time of about 5–7 min [2]. Finally, the commands used are easy for patients to understand, and thanks to the accompanying illustrations, the tool is also attractive and accessible to children. Hence, it can find application among even the youngest groups of patients.

## 5. Conclusions

The Polish version of the Nordic Orofacial Test-Screening is linguistically understandable and culturally adapted for children, adolescents, and adults. The results of this study indicate that orofacial function screening is advisable, and orofacial dysfunctions and parafunctions are frequent in the healthy population.

## Figures and Tables

**Table 1:** Translation discrepancies detected during the validation process.

Original English	Polish Translation	Back-Translation	Corrected Polish Version
manual	instrukcja	instruction	przewodnik
drooling	ślinienie	salivation	nadmierne ślinienie
support	środki wspomagające	aids	aparatura

**Table 2:** Translation equivalents detected during the validation process.

Equivalence	Original NOT-S	Back-Translation	Polish Translation
Semantic	developed	designed	opracowany
	form	assessment form	formularz
	web shop	web store	w sklepie internetowym
	anamnestic section	objective examination	badanie podmiotowe
	reply	answer	odpowiedź
	explains	clarifies	wyjaśnia
	conducted	carried out	przeprowadzane
	elicit	trigger	wywołuje
	forefinger	index	palca wskazującego
Experiential	interview	examination	wywiad
	jaw	mandibular	żuchwa
	physician	medical doctor	lekarz
	obvious	marked	wyraźny
	queasiness	nausea	mdłości
	refusal	reflux	refluks
	cpap	cpap machine	aparat cpap
	respirator	ventilator	respirator
	outside of the Vermillion border	beyond the lip red	poza czerwień wargową ust
Conceptual	phone	calling	telefonicznie
	via	from the	w
	from 3 years	for 3-year-olds and above	od 3. roku życia
	difficulties to speak, chew or swallow	difficulty speaking, chewing or swallowing	trudności z mówieniem, żuciem lub połykaniem
	contains	consists of	składa się z
	sections	parts	części
	dryness of the mouth	dry mouth	suchość w jamie ustnej
	nose	nasal	nos
	specify	indicate	podaj
	code	classification	kod
	seated	sitting	siedząca
	lying down	supine	leżąca
	position	positioning	w pozycji
	normal	neutral	neutralne
	straight	vertical	wyprostowane
	score	number	liczba
	can vary	can range	może wynosić
	furthest to the right	the rightmost	najbardziej wysuniętej w prawo
	becomes	is	–
	mouth	oral cavity	jama ustna
	decreased	reduced	obniżona
	much	often	często
	when	while	podczas
	sleep	sleeping	snu
	allergies	allergy	alergia
	bite hard	bite down	zaciskasz zęby
	eat	intake food	przyjmuje
	with the mouth	orally	doustnie
	find it	have	masz
	to eat	eating	spożywaniem
	consistencies	consistency	konsystencji
	exclude	excluding	z wyłączeniem
	-	your	ci
	-	them	ich
	get	appear	pojawia się
	does not apply	irrelevant	nieistotne
	sore	hurt	dolegliwości bólowe
	apply	involve	dotyczy
	watch	observe	obserwuj
	picture	figure	ilustrację
	starting	begin	zaczynając od
	-	for	trwa
	assess	grades	oceń
	deviant	incorrect	nieprawidłowe
	position	positioning	ułożenie
	in the face	facial	twarzy
	smell	sniff	powąchaj
	succession	consecutive	kolejnych
	can manually	may help his hands	może użyć rąk
	not activated	not work	nie pracują
	easily	clearly	wyraźne
	cannot pout	unable	nie potrafi
	no marked	no clear	nie można
	registered	observed	zaobserwować
	wet	moisten	zwilżenia
	blow up	puff up	nadmij policzki
	air leaking out	expelling air	wypływu powietrza
	no marked	no obvious	nie widać
	unclear	slurred	niewyraźna
	indistinct	unclear	niewyraźnymi
Other discrepancies	used	to be used	do stosowania

**Table 3:** Baseline characteristics of the participants.

Sample Characteristics		*n*	%
Sample count		50	100
Sex	female male	30 20	60 40
Age	min max mean SD	3 34 15.8 8.9	
Answers with third-person assistance		24	48
Total NOT-S score	min max	0 5	
Interview score	min max	0 3	
Examination score	min max	0 2	

**Table 4:** Distribution of total NOT-S scores of the cultural adaptation.

The Total NOT-S Scores	Frequencies
0.0	16%
1.0	32%
2.0	36%
3.0	8%
4.0	6%
5.0	2%

**Table 5:** Distribution of NOT-S scores in each section and part.

Part	Name of Section	Question	Each Question		Whole Domain		*p*
			*n*	%	*n*	%	
Interview	I Sensory function	I A Does brushing your teeth trigger a gag reflex?	1	2	7	14	0.70913
		I B Do you put so much food in your mouth that it is difficult to chew?	6	12			
	II Breathing	II A Do you use any breathing aids?	0	0	6	12	-
		II B Do you often snore while sleeping?	6	12			
	III Habits	III A Do you bite your nails, suck your fingers or other objects every day?	18	36	39	78	0.87087
		III B Do you suck or bite your lips, tongue or cheeks every day?	22	44			
		III C Do you bite or grind your teeth a lot during the day?	18	36			
	IV Chewing and swallowing	IVA Does not intake food orally	0	0	13	26	0.00073 *
		IV B Do you have difficulty eating foods of a certain consistency?	4	8			
		IV C Does it take you 30 min or more to eat your main meal?	4	8			
		IV D Do you swallow large bites without chewing them?	6	12			
		IV E Do you often cough during meals?	2	4			
	V Drooling	V A Does saliva appear in the corner of your mouth or on the chin almost every day?	3	6	3	6	-
	VI Dryness of the mouth	VI A Do you have to drink something to eat a cracker?	6	12	7	14	0.09144
		VI B Does your mouth or tongue hurt?	2	4			
Examination	1 Face at rest	1 A Asymmetry	0	0	1	2	-
		1 B Incorrect lip positioning	0	0			
		1 C Incorrect tongue positioning	0	0			
		1 D Involuntary movements	1	2			
	2 Nose breathing,	2 A Close your mouth and take 5 deep breaths through your nose (sniff).	0	0	0	0	-
	3 Facial expression	3 A Close your eyes tightly.	1	2	3	6	0.00006 *
		3 B Show your teeth.	3	6			
		3 C Try to whistle (blow).	0	0			
	4 Masticatory muscle and jaw function	4 A Bite down hard on the back teeth.	7	14	7	14	-
		4 B Open your mouth as wide as you can.	0	0			
	5 Oral motor function	5 A Stick your tongue out as far as you can.	1	2	1	2	-
		5 B Lick your lips.	0	0			
		5 C “Puff up your cheeks”, hold for at least 3 s	0	0			
		5 D Open your mouth wide and say ah, ah, ah!	0	0			
	6 Speech	6 A Doesn’t speak.	0	0	0	0	-
		6 B Count to ten out loud.	0	0			
		6C Say pataka-pataka-pataka.	0	0			

* Significance level *p* ≤ 0.05; The chi-square test was used; *n*—number; %—percentage.

## Data Availability

The article encompasses the original contributions outlined in the study. Additional enquiries may be sent to the corresponding author.

## Supplementary Materials

The following supporting information can be downloaded at: https://www.mdpi.com/article/10.3390/diagnostics15131656/s1, Nordic Orofacial Test-screening.

## Abbreviations

The following abbreviations are used in this manuscript:

NOT-S	Nordic Orofacial Test-Screening
RDC/TMD	Research Diagnostic Criteria for Temporomandibular Disorders
