# Supplementary material for: Nordic Orofacial Test-Screening Protocol as a Tool for Assessment of Orofacial Dysfunction in Pediatric and Adult Patients
Source: Diagnostics (Basel). 2025 Jun 29;15(13):1656. doi: 10.3390/diagnostics15131656 (PMC12249170; doi:10.3390/diagnostics15131656)
Supplement: Supplementary file 1 [file diagnostics-15-01656-s001.zip › NOT-S- PL.pdf]

Nordycki Test przesiewowy do oceny funkcji ustno-twarzowej

NOT-S

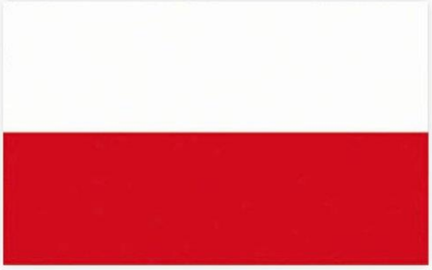

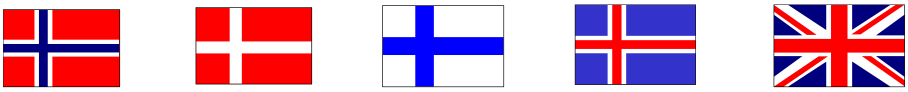

NOT-S został opracowany przez Merete Bakke, Copenhagen; Birgittę Bergendal, Jönköping; Anitę McAllister, Linköping; Lottę Sjögreen, Göteborg; and Pamelę Åsten, Oslo; przy wsparciu Nordic Association for Disability and Oral Health, NFH (Nordyckiego Stowarzyszenia na rzecz Osób Niepełnosprawnych i Zdrowia Jamy Ustnej).

Ten formularz można pobrać ze strony internetowej www.mun-h-center.se.
Do stosowania jest z ilustrowanym przewodnikiem, który można zamówić w sklepie internetowym Mun-H-Center lub telefonicznie pod numerem +46 31 750 92 00.

© 2007 NFH

Nordycki Test przesiewowy do oceny funkcji ustno-twarzowej NOT-S *– (od 3. roku życia)*

NOT-S jest stosowany, gdy pacjent ma trudności z mówieniem, żuciem lub połykaniem. Badanie podmiotowe jest przeprowadzane w formie ustrukturyzowanego wywiadu. Osoba przeprowadzające badanie zadaje pytania, wyjaśnia, a w razie potrzeby zadaje dodatkowe pytania, interpretuje odpowiedź i wypełnia formularz.

Wywiad NOT-S składa się z sześciu części: funkcje sensoryczne, oddychanie, nawyki, żucie i połykanie, nadmierne ślinienie oraz suchość w jamie ustnej (I–VI).

Badanie NOT-S składa się sześciu części: twarz w spoczynku, oddychanie przez nos, wyraz twarzy, funkcja żuchwy i mięśni żucia, funkcje motoryczne jamy ustnej oraz mowa (1–6).

Podczas badania należy korzystać z ilustrowanego przewodnika.

Kraj PL ☐ EN ☐ NO ☐ SE ☐ SF ☐ inny ☐

Osoba badająca logopeda ☐ lekarz dentysta ☐ lekarz ☐ fizjoterapeuta ☐ inny ☐

Data badania |____|____|____|____|____|____|____|____| rok miesiąc dzień

Data urodzenia	|____|____|____|____|____|____|____|____| rok miesiąc dzień ♀ ☐ ♂ ☐
Imię nazwisko/ID	

Podstawowa diagnoza medyczna (podaj tylko jedną) ______________________

Kod diagnostyczny (ICD-10): ______________________

Pozycja podczas badania ☐ siedząca ☐ leżąca

Ułożenie głowy w pozycji siedzącej ☐ neutralne (pionowe i wyprostowane) ☐ inna

Odpowiedzi z pomocą innej osoby ☐

**Kod badania przesiewowego**<br><br>**Całkowita liczba punktów NOT-S może wynosić od 0 do 12**	**X = tak<br>0 = nie<br>– = nie poddano ocenie**	**Jeśli w danej części znajduje się jedna lub więcej odpowiedzi X, w kratce najbardziej wysuniętej na prawo od niej należy wpisać ocenę 1.**
NOT-S	***Całkowita liczba punktów*** ☐☐	

 © *2007 NFH*

Wywiad NOT-S

		WYNIK
I	*Funkcje sensoryczne*<br>*A.* **Czy mycie zębów wywołuje odruch wymiotny? Czy zdarza się to prawie za każdym razem?** ☐<br>*Opis: Wyraźny dyskomfort taki jak mdłości, wymioty lub refluks (zwiększona wrażliwość).*<br>B. **Czy wkładasz do ust tak dużo jedzenia, że trudno je przeżuwać? Czy zdarza się to codziennie?** ☐<br>*Opis*: Nie wie, kiedy jama ustna jest wypełniona (obniżona wrażliwość).	☐
II	*Oddychanie*<br>*A.* **Czy używasz jakiejkolwiek aparatury wspomagającej oddychanie?** ☐<br>*Opis: aparat CPAP, respirator, tlen, inne.*<br>**B. Czy często chrapiesz podczas snu? Czy dzieje się tak prawie każdej nocy?** ☐<br>*Opis: Chrapanie lub bezdech. Nie dotyczy objawów astmy lub alergii.*	☐
III	Nawyki<br>**A. Czy codziennie obgryzasz paznokcie, ssiesz palce lub inne przedmioty?** ☐<br>*Opis: Używanie smoczka i ssanie palców nie jest oceniane poniżej 5. roku życia.*<br>**B. Czy codziennie ssiesz lub przygryzasz wargi, język lub policzki?** ☐<br>**C. Czy w ciągu dnia mocno zagryzasz zęby lub zgrzytasz zębami?** ☐	☐
IV	*Żucie i połykanie*<br>**A. Nie przyjmuje pokarmów doustnie.** ☐<br>***(sonda nosowo-żołądkowa, gastrostomia lub inne). Pomiń pytania B–E.***<br>**B. Czy masz trudność ze spożywaniem pokarmów o określonej konsystencji?** ☐<br>*Opis: Z wyłączeniem alergii i specjalnych diet, takich jak* wegetariańska, wegańska, bezglutenowa.<br>**C. Czy zjedzenie głównego posiłku zajmuje Ci 30 minut lub więcej?**<br>**D. Czy połykasz duże kęsy nie przeżuwając ich?** ☐<br>**E. Czy często kaszlesz podczas posiłków?** ☐<br>*Opis. Zdarza się to prawie przy każdym posiłku.* ☐	☐
V	*Nadmierne ślinienie*<br>*A.* **Czy ślina pojawia się w kąciku ust lub na brodzie prawie codziennie?** ☐<br>Opis*: Musi wytrzeć usta. Nieistotne podczas snu.*	☐
VI	*Suchość w jamie ustnej*<br>**A. Czy musisz popić żeby zjeść krakersa?** ☐<br>**B. Czy odczuwasz dolegliwości bólowe ust lub języka?** ☐<br>*Opis: Nawracający ból lub uczucie pieczenia co najmniej raz w tygodniu.*<br>***Nie dotyczy bólu zęba ani pęcherzyków (zmiany przypominające pęcherze) w jamie ustnej.***	☐
Imię i nazwisko/ID:	*Badanie NOT-S* *Suma*	☐

© 2007 NFH

Badanie NOT-S

		WYNIK
**1.**	***Twarz w spoczynku*** Obserwuj ilustrację przez minutę, rozpoczynając od teraz.<br>Ilustracja 1 *Obserwacja trwa 1 minutę. Oceń A-D.*<br>***A.* Asymetria.** ☐<br>*Opis:* *Dotyczy zarówno szkieletu, jak i tkanek miękkich.*<br>***B.* Nieprawidłowe ułożenie warg.** ☐<br>*Opis:* *Otwarte usta lub inne nieprawidłowości przez ponad 2/3 czasu.*<br>***C.* Nieprawidłowe ułożenie języka.** ☐<br>*Opis:* *Koniec języka widoczny pomiędzy zębami przez ponad 2/3 czasu.*<br>***D.* Ruchy mimowolne.** ☐<br>*Opis:* *Powtarzające się ruchy mimowolne twarzy.*	☐
**2.**	***Oddychanie przez nos***<br>Ilustracja 2 ***A.* Zamknij usta i weź 5 głębokich oddechów przez nos (powąchaj).**<br>***Kryterium:*** *Nie jest w stanie wziąć 5 kolejnych oddechów przez nos.*<br>*Jeśli pacjent nie może zamknąć ust, może użyć rąk albo skorzystać z pomocy osoby badającej.* ☐<br>***Nie podlega ocenie gdy pacjent jest przeziębiony.***	☐
**3.**	***Wyraz twarzy***<br>Ilustracja 3 ***A.* Zamknij mocno oczy.** ☐<br>***Kryterium:*** *Mięśnie twarzy nie pracują symetrycznie.*<br>Ilustracja 4 **B. Pokaż zęby.** ☐<br>***Kryterium:*** *Przy wyraźnym uwidocznieniu zebów praca mięśni warg i twarzy nie jest symetryczna.* ☐<br>Ilustracja 5 ***C.* Spróbuj zagwizdać (dmuchnąć).**<br>***Kryterium:*** *Nie potrafi symetrycznie zaokrąglić I wydąć ust.*	☐
**4.**	***Funkcja żuchwy i mięśni żucia***<br>Ilustracja 6 ***A.* Nagryź mocno na tylne zęby.** ☐<br>**Kryterium:** *Nie można zaobserwować wyraźnej symetrycznej aktywności mięśni żwaczy po obu stronach podczas badania dwoma palcami.*<br>Ilustracja 7 ***B.* Otwórz usta tak szeroko, jak tylko potrafisz.** ☐<br>***Kryterium:*** *Nie może otworzyć ust na szerokość palca wskazującego i środkowego, lewej ręki pacjenta.*<br>***W przypadku braku przednich zębów należy zastosować miarę szerokości trzech palców (wskazującego, środkowego i serdecznego).***	☐
**5.**	***Funkcje motoryczne jamy ustnej***<br>Ilustracja 8 ***A.* Wystaw język tak daleko jak możesz.** ☐<br>***Kryterium:*** *Nie może sięgnąć końcem języka poza czerwień wargową ust.*<br>Ilustracja 9 **B. Obliż usta.** ☐<br>***Kryterium:*** *Nie może użyć końca języka do zwilżenia ust i nie sięga do ich kącików.*<br>Ilustracja 10 ***C.*"Nadmij policzki",przytrzymaj przez co najmniej 3 sekundy.**<br>***Kryterium:*** *Nie może nadąć policzków bez wypływu powietrza lub bez wydawania dźwięków.* ☐<br>Ilustracja 11 **D. Otwórz szeroko usta i powiedz A, A, A!**<br>***Kryterium:*** *Nie widać wyraźnego uniesienia języczka i podniebienia miękkiego.* ☐	☐
**6.**	***Mowa*** ***A.* Nie mówi.** Pomiń zadania *B-C.* ☐<br>Ilustracja 12 ***B.* Policz na głos do dziesięciu.** ☐<br>***Kryterium:*** *Mowa jest niewyraźna z jednym lub kilkoma niewyraźnymi dźwiękami lub nieprawidłowym rezonansem nosowym. Poniżej 5 roku życia wyłączamy dźwieki R, CZ, Ż, SZ, DŻ z oceny.*<br>Ilustracja 13 **C. Powiedz pataka-pataka-pataka.** ☐<br>***Kryterium:*** *Nie podlega ocenie u dzieci poniżej 5. roku życia.*	☐

Imię i nazwisko/ID: ***Badanie NOT-S*** ***Suma*** ☐

 © 2007 NFH
