# Supplementary material for: Nordic Orofacial Test-Screening Protocol as a Tool for Assessment of Orofacial Dysfunction in Pediatric and Adult Patients
Source: Diagnostics (Basel). 2025 Jun 29;15(13):1656. doi: 10.3390/diagnostics15131656 (PMC12249170; doi:10.3390/diagnostics15131656)
Supplement: Supplementary file 1 [file diagnostics-15-01656-s001.zip › not-s-manual_eng_090625.pdf]

Nordic Orofacial Test - Screening

NOT-S

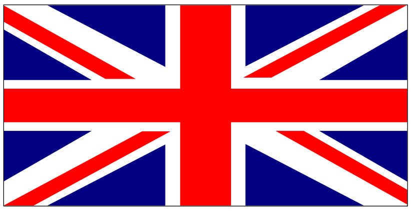

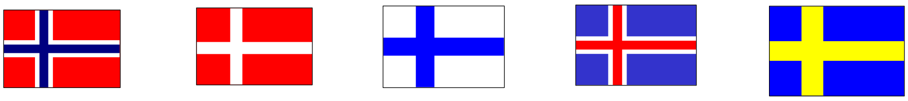

NOT-S was developed by Merete Bakke, Copenhagen; Birgitta Bergendal, Jönköping; Anita McAllister, Linköping; Lotta Sjögreen, Göteborg; and Pamela Åsten, Oslo; with the support of the Nordic Association for Disability and Oral Health, NFH.

This assessment form can be downloaded from www.mun-h-center.se.
To be used with the illustrated manual that can be ordered via Mun-H-Center's web shop or phone +46 31 750 92 00.

 © *2007 NFH*

Nordic Orofacial Test NOT-S *– screening (from 3 years)*

NOT-S is used when a patient has difficulties to speak, chew, or swallow.
The anamnestic section is conducted as a structured interview. The examiner asks a question, explains, and asks additional questions when necessary, interprets the reply, and fills in the form.

The NOT-S interview contains six sections: sensory function, breathing, habits, chewing and swallowing, drooling, and dryness of the mouth (I–VI).

NOT-S examination contains six sections: face at rest, nose breathing, facial expression, masticatory muscle and jaw function, oral motor function, and speech (1–6).

The illustrated manual is to be used during the examination.

Country DK ☐ IS ☐ NO ☐ SE ☐ SF ☐ Other ☐ ______________________

Examiners Speech therapist ☐ Dentist ☐ Physician ☐ Physio therapist ☐ Other ☐ ______________________

Date of examination |____|____|____|____|____|____|____|____| y y y y m m d d

Date of birth |____|____|____|____|____|____|____|____| y y y y m m d d ♀ ☐ ♂ ☐

Name/ID

*Primary medical diagnosis (specify only **one**)* ______________________

Diagnostic code (ICD-10): ______________________

Examination position ☐ seated ☐ lying down

Position of the head when seated ☐ normal (upright and straight) ☐ other

Answers with the help of another person ☐

Code for screening<br><br>**The** NOT-S total score can vary from 0 to 12	**X = yes**<br>**0 = no**<br>**– = not assessed**	**If there are one or more X answers in a section, place a score of 1 in the box furthest to the right.**

NOT-S ***Total score*** ☐☐

 © 2007 NFH

NOT-S interview

					SCORE
I	***Sensory function***				
		A.	Does brushing your teeth elicit a gag reflex?<br>Does this happen almost every time?	☐	
	Description:		*Obvious discomfort such as queasiness, vomiting, or refusal (increased sensitivity).*		
		B.	Do you put so much food in your mouth that it becomes difficult to chew?<br>Does this happen every day?	☐	
	Description:		*Doesn't know when the mouth is full (decreased sensitivity).*		☐
II	***Breathing***				
		A.	Do you use any breathing support?	☐	
	Description:		*CPAP, respirator, oxygen, other.*		
		B.	Do you snore much when you sleep?<br>Does this happen almost every night?	☐	
	Description:		*Snoring or apnoea. Does not apply to symptoms from asthma or allergies.*		☐
III	***Habits***				
		A.	Do you bite your nails, or suck your fingers, or other objects every day?	☐	
	Description:		*Use of a pacifier and sucking on the fingers is not assessed under 5 years of age.*		
		B.	Do you suck or bite your lips, your tongue, or your cheeks every day?	☐	
		C.	Do you bite your teeth together hard or grind your teeth during the day?	☐	☐
IV	***Chewing and swallowing***				
		A.	Does not eat with the mouth *(nasogastric tube, gastrostomy or other)*.<br>*Skip question B–E.*	☐	
		B.	Do you find it difficult to eat foods with certain consistencies?	☐	
	Description:		*Exclude allergies and special diets such as vegetarian, vegan, and gluten-free.*		
		C.	Does it take you 30 minutes or more to eat a main meal?	☐	
		D.	Do you swallow large bites without chewing?	☐	
		E.	Do you often cough during meals?	☐	
	Description:		*It happens at almost every meal.*		☐
V	***Drooling***				
		A.	Do you get saliva in the corner of your mouth or on your chin almost every day?	☐	
	Description:		*Needs to wipe their mouth. Does not apply during sleep.*		☐
VI	***Dryness of the mouth***				
		A.	Do you have to drink to be able to eat a cracker?	☐	
		B.	Do you have sore mouth or a sore tongue?	☐	
	Description:		*Recurrent pain or burning sensation at least once a week.*<br>*Does not apply to toothache or vesicles (blister-like lesions) in the mouth.*		☐
Name/ID:			***NOT-S interview***	***Sum***	☐

© 2007 NFH

NOT-S examination

SCORE

1 *Face at rest*

Picture 1 — Watch the picture for one minute. Starting now.
Observation for a total of 1 minute. Assess A-D.

A. Asymmetry ☐
Description: *Concerns both the skeleton and soft tissues.*

B. Deviant lip position ☐
Description: *Open mouth or other deviations more than 2/3 of the time.*

C. Deviant tongue position ☐
Description: *Tip of the tongue visible between the teeth more than 2/3 of the time.*

D. Involuntary movements ☐
Description: *Repeated involuntary movements in the face.* ☐

2 *Nose breathing*

Picture 2 *A.* Close your mouth and take 5 deep breaths through your nose (smell) ☐
Criterion: *Is unable to take 5 breaths in succession through the nose.*
If the patient cannot close their lips, the patient or the examiner can manually help the lips to close. Do not assess if the patient has a cold. ☐

3 *Facial expression*

Picture 3 *A.* Close your eyes tightly ☐
Criterion: *The facial muscles are not activated in a strongly symmetrical fashion.*

Picture 4 *B.* Show your teeth ☐
Criterion: *The lip and facial muscles are not symmetrically activated so that the teeth are easily visible.*

Picture 5 *C.* Try to whistle (blow) ☐
Criterion: *Cannot pout and round the lips symmetrically.* ☐

4 *Masticatory muscle and jaw function*

Picture 6 *A.* Bite hard on your back teeth ☐
Criterion: *No marked symmetrical activity can be registered when two fingers are held on the jaw muscles (the musculus masseter on both sides).*

Picture 7 *B.* Open your mouth as wide as you can ☐
Criterion: *Cannot open their mouth a distance corresponding to the width of the forefinger and the middle finger on the patient's left hand. If the front teeth are missing, use a three-finger width (the forefinger, and the middle and ring fingers) as a measure.* ☐

5 *Oral motor function*

Picture 8 *A.* Stick out your tongue as far as you can ☐
Criterion: *Cannot reach outside of the Vermillion border of the lips with the tip of the tongue.*

Picture 9 *B.* Lick your lips ☐
Criterion: *Cannot use the tip of the tongue to wet the lips and cannot reach the corners of the mouth.*

Picture 10 *C.* "Blow up" your cheeks and hold for at least 3 seconds ☐
Criterion: *Cannot "blow up" the cheeks without air leaking out or without making sounds.*

Picture 11 *D.* Open your mouth wide and say ah, ah, ah [a]! ☐
Criterion: *No marked elevation of the uvula and the soft palate can be observed.* ☐

6 *Speech*

A. Does not speak. *Skip task B-C.* ☐

Picture 12 *B.* Count out loud to ten ☐
Criterion: *Speech is unclear with one or more indistinct sounds or abnormal nasality.*
Under 5 years of age, exclude R, S, and TH sounds from the assessment.

Picture 13 *C.* Say pataka-pataka-pataka ☐
Criterion: *Do not assess this in children under 5 years of age.* ☐

Name/ID: ***NOT-S examination*** ***Sum*** ☐

© 2007 NFH
